# A Multicenter Study to Evaluate Harmonization of Assays for C-Terminal Telopeptides of Type I Collagen (ß-CTX): A Report from the IFCC-IOF Committee for Bone Metabolism (C-BM)

**DOI:** 10.1007/s00223-021-00816-5

**Published:** 2021-03-04

**Authors:** E. Cavalier, R. Eastell, N. R. Jørgensen, K. Makris, S. Tournis, S. Vasikaran, J. A. Kanis, C. Cooper, H. Pottel, H. A. Morris, on behalf of the IFCC-IOF Committee for Bone Metabolism (C-BM)

**Affiliations:** 1 Department of Clinical Chemistry, University of Liège, CHU Sart-Tilman, Domaine du Sart-Tilman, B-4000 Liège, Belgium; 2 Mellanby Centre for Bone Research, University of Sheffield, Sheffield, UK; 3 Department of Clinical Biochemistry, Rigshospitalet, 2600 Glostrup, Denmark; 4 Department of Clinical Medicine, Faculty of Health and Medical Sciences, University of Copenhagen, Copenhagen, Denmark; 5 Clinical Biochemistry Department, KAT General Hospital, 14561 Athens, Greece; 6 Laboratory for Research of the Musculoskeletal System “Th. Garofalidis”, Medical School, University of Athens, 14561 Athens, Greece; 7 PathWest Laboratory Medicine, Fiona Stanley Hospital, Murdoch, WA 6150 Australia; 8 Centre for Metabolic Bone Diseases, University of Sheffield Medical School, Beech Hill Road, Sheffield, S10 2RX UK; 9 The MRC Epidemiology Resource Centre, Southampton General Hospital, University of Southampton, Southampton, SO16 6YD UK; 10 Department of Public Health and Primary Care, KU Leuven Campus Kulak Kortrijk, Kortrijk, Belgium; 11 School of Pharmacy and Medical Sciences, University of South Australia, Adelaide, SA 5000 Australia; 12 Mary McKillop Institute for Health Research, Australian Catholic University, Melbourne, Australia

**Keywords:** C-terminal telopeptide of type I collagen, ß-crosslaps, ß-CTX, Bone turnover markers, Bone resorption, Harmonization

## Abstract

**Background:**

Biochemical bone turnover markers are useful tools to assess bone remodeling. C-terminal telopeptide of type I collagen (ß-CTX) has been recommended as a reference marker for bone resorption in research studies.

**Methods:**

We describe the results of a multicenter study for routine clinical laboratory assays for ß-CTX in serum and plasma. Four centers (Athens GR, Copenhagen DK, Liege BE and Sheffield UK) collected serum and plasma (EDTA) samples from 796 patients presenting to osteoporosis clinics. Specimens were analyzed in duplicate with each of the available routine clinical laboratory methods according to the manufacturers’ instructions. Passing-Bablok regressions, Bland–Altman plots, V-shape evaluation method, and Concordance correlation coefficient for ß-CTX values between serum and plasma specimens and between methods were used to determine the agreement between results. A generalized linear model was employed to identify possible variables that affected the relationship between the methods. Two pools of serum were finally prepared and sent to the four centers to be measured in 5-plicates on 5 consecutive days with the different methods.

**Results:**

We identified significant variations between methods and between centers although comparison results were generally more consistent in plasma compared to serum. We developed univariate linear regression equations to predict Roche Elecsys®, IDS-iSYS, or IDS ELISA ß-CTX results from any other assay and a multivariable model including the site of analysis, the age, and weight of the patient. The coefficients of determination (R^2^) increased from approximately 0.80 in the univariate model to approximately 0.90 in the multivariable one, with the site of analysis being the major contributing factor. Results observed on the pools also suggest that long-term storage could explain the difference observed with the different methods on serum.

**Conclusion:**

Our results show large within- and between-assay variation for ß-CTX measurement, particularly in serum. Stability of the analyte could be one of the explanations. More studies should be undertaken to overcome this problem. Until harmonization is achieved, we recommend measuring ß-CTX by the same assay on EDTA plasma, especially for research purposes in large pharmacological trials where samples can be stored for long periods before they are assayed.

## Introduction

Determination of C-terminal telopeptide (β-CTX) and N-terminal propeptide (PINP) of type I procollagen as reference markers of bone resorption and formation, respectively, is recommended analytes since 2010 by the International Osteoporosis Foundation (IOF) and the International Federation of Clinical Chemistry and Laboratory Medicine (IFCC) Joint Working Group on Bone Marker Standards (WG-BMS) [1]. As a result, these markers are now recommended by IOF, the European Calcified Tissue Society (ECTS) and the Society for Clinical and Economic Aspects of Osteoporosis, Osteoarthritis and Musculoskeletal Diseases (ESCEO) as useful tools for monitoring adherence of patients with osteoporosis to therapy with oral bisphosphonates [2, 3]. One of the major issues that impede the implementation of these bone turnover markers (BTMs) in clinical practice is the lack of standardization between the different methods available on the market. For PINP, we have recently shown that the two automated methods, IDS-iSYS (Boldon, UK), and Roche Elecsys® total P1NP assay run on cobas e family instruments (Mannheim, Germany) provided results that could be used interchangeably, allowing the application of similar reference ranges whatever the method, which is an important finding for the routine use of biomarkers outside the United States [4, 5]. Indeed, in that country, the only approved method is the Orion UniQ manual radio-immunoassay (Espoo, Finland), which presents a significant proportional bias compared to the two automated methods.

β-CTX can also be measured by two automated methods, IDS-iSYS and Roche Elecsys® β-CrossLaps assay run on cobas e family instruments, as well as with a manual ELISA also marketed by IDS. Previous studies have, however, shown that there was significant disagreement between the results generated from patient samples by these three β-CTX assays [6]. Hence, in order to establish the clinical value of β-CTX as a reference bone resorption biomarker, harmonization of the results from different assays for these biomarkers is necessary. The IFCC and IOF established a Joint Working Group for the Standardization of Bone Marker Assays (WG-BMA) in 2012, which became a Joint Committee in 2019 to, among other tasks, evaluate the harmonization of β-CTX assays. In this study, we report the results of the comparison of β-CTX results generated by each of the available routine clinical assays on the samples of the patients enrolled in the multicenter study for which we have already reported the results for PINP [5].

## Material and Methods

### Patients and Samples

The characteristics and the protocol of this study have already been described elsewhere [5]. Briefly, four centers located in Athens (Greece, GR), Copenhagen (Denmark, DK), Liege (Belgium, BE), and Sheffield (United Kingdom, UK) took part in the study. According to the agreed protocol, each center recruited 200 patients attending a local osteoporosis clinic. Patient blood samples were collected in K_3_-EDTA tubes for analyses on plasma and in tubes with gel for analyses on serum. Demographic data, including sex, age, fasting status, current medications, and bone mineral density (BMD), were also collected.

**Fig. 1:**
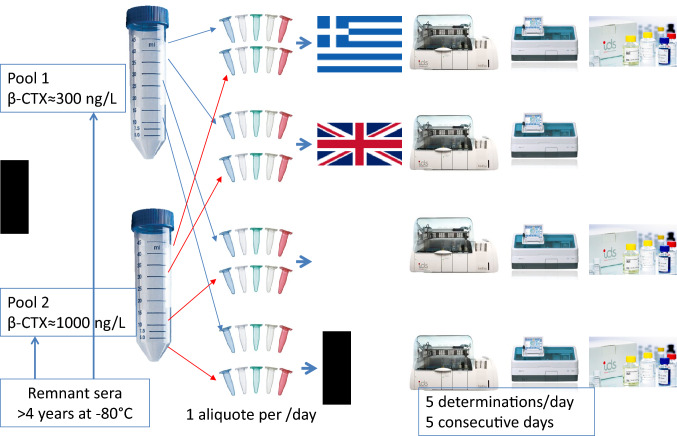
Preparation of the pools for the performance study evaluation

Simultaneously, each center was asked to run 2 serum pools in 5 replicates over 5 consecutive days, on each instrument without recalibration according to CLSI EP15-A3 [7]. This performance study was run in parallel with the measurements of the patients’ samples. The two serum pools were constituted in BE using remnant human samples that had been stored at − 80 °C for > 5 years. These samples were mixed together according to their original value to target a final β-CTX concentration of ≈300 and ≈1000 ng/L. After thorough homogenization for 1 h on a rotating plate, pools were centrifuged and aliquoted. The aliquots were stored at − 80 °C until shipment on dry ice to each participating center. Each center, thus, received 5 aliquots (1 for each day) for each instrument (Fig. 1).

### Analytical Methods

The two in-vitro diagnostics (IVD) companies producing β-CTX assay kits (IDS and Roche) provided reagents and calibrators to the participating laboratories. All reagents were from the same lot, and a single calibration was used (except for the ELISA). IDS provided reagents for the iSYS analyzer and ELISA kits whereas Roche provided reagents for the **cobas e** 411 analyzer which was the instrument used in all centers. All the laboratories had previous experience in running these methods and all assays were run in all laboratories, except IDS ELISA which was not run in the UK. Manufacturers claim that β-CTX can be measured either in serum or plasma. Hence, plasma and serum samples were run in duplicate according to the manufacturers’ instructions, and results were calculated if the standard curves and the manufacturers’ supplied internal quality control (QC) specimens were within the specifications.

### Statistical Methods

MedCalc (Mariakerke, BE) was used to calculate the Passing-Bablok regressions between serum and plasma and between methods as well as Bland–Altman plots. The coefficients of variations were calculated on duplicates to determine the repeatability of each assay. The mean of the duplicates was used to compare the results. The Mann–Whitney test was used to compare the medians.

The familiarization panel was analyzed per level using an ANOVA model accounting for the effects of center, day, and the interaction between day and center, and the QC results between centers were compared with ANOVA, followed by Tukey’s multiple comparison test with the 95% confidence intervals method (Graphpad Prism 6).

Because the agreement between results indicated considerable deviation from the ideal situation (slope ≠ ‘1’, intercept ≠ ‘0’, R^2^ ≠ 1), the GLMSELECT procedure, using the backward selection option in SAS 9.4 (SAS Institute Inc., Cary, NC, USA), was applied to establish a generalized linear model (GLM) for each comparison with the aim of identifying variables which affected the differences between methods and, ultimately, to obtain a more acceptable prediction model. GLMSELECT provides *t* values for the coefficients (instead of *p* values). An absolute *t* value > 1.96 corresponds with a *p* value < 0.05. The larger the absolute *t* value, the more important the coefficient in the GLM.

Since small deviations from the ideal situation will be statistically significant because of the (very) large sample size of this study, we defined specifications corresponding to the desirable bias of 12.6% based on β-CTX biological variation according to Cavalier et al. [8] for the slope and ± 50 ng/L for the intercept corresponding to the limit of quantification of the assays to build V-shape limits (defined as *y* = − 0.126 *x* − 50 and *y* = 0.126 *x* + 50) for the regression of differences on averages. We calculated the percentages of samples comprised between the V-shape limits and considered that methods were equivalent if 90% of the samples were comprised within the limits.

Finally, we also used Mecalc to calculate the concordance correlation coefficient (CCC), which evaluates the degree to which pairs of observations fall on the 45° line through the origin according to Lin et al. [9] and the strength of agreement according to Mc Bride et al. [10] as well as the Pearson correlation coefficient (*ρ*), which measures how far each observation deviates from the best-fit line and is, thus, a measure of precision, and a bias correction factor (*C*_b_) that measures how far the best-fit line deviates from the 45° line through the origin and is, thus, a measure of accuracy. The strength of agreement is considered as “poor” when the CCC is < 0.90, “moderate” when CCC is comprised between 0.90 and 0.95, “substantial” between 0.95 and 0.99 and “almost perfect” when CCC is > 0.99.

## Results

All the calibration curves were accepted by the instruments, and all the QCs were within the specifications provided by the manufacturers in all participating centers.

### Patients

796 patients (692 women, 104 men) were included in the study. Mean age (± SD) was 66.1 (± 11.7) years and mean BMI was 25.9 (± 4.8) kg/m^2^. There were mean age differences between patients recruited at the various centers: GR had the youngest patient group (mean age = 61.6 ± 8.8) and DK had the oldest patient group (mean age = 70.1 ± 11.3). All patients were in the fasting state in BE and GR whereas this was not necessarily the case in DK and UK. Samples were collected between April 2014 and April 2016 in BE, between April 2016 and November 2016 in UK, April 2015 and March 2016 in DK and between October and December 2016 in GR. All samples were stored at − 80 °C until determination, which occurred between December 2016 and January 2017 in all four centers.

Regarding treatment, 65.8% of patients were taking calcium, 60.8% vitamin D, 11.1% vitamin D analogs, 25.9% bisphosphonates, 0.3% strontium ranelate, 9.0% denosumab, 2.0% teriparatide, and 1.1% were treated by selective estrogen receptors modulators.

### Results of the Performance Evaluation Study

#### Analysis of the Results

Mean of pools were quite different according to the methods: 173.9 ± 19.7 ng/L and 763 ± 102 ng/L for the Elecsys assay on levels 1 and 2, respectively, vs. 81.6 ± 12.7 ng/L and 650 ± 59 ng/L for iSYS and 168 ± 32 and 737 ± 162 ng/L for ELISA.

For Roche, mean values were similar for BE, DK, and UK but consistently lower for GR, and the standard deviations in GR were consistently greater each day. On day 5, the mean in UK was significantly lower than on all other days in BE and DK. The ANOVA model explained 92.6% of the variation in the results with center, day, and the interaction between center and day accounting for 78.2%, 3.4%, and 10.9% of that variation, respectively. Similar findings were obtained for pool 2, but in this case, on day 4, DK showed a very large difference between the individual results.

For iSYS, distribution of the values was normal, but mean values per center were also quite different, ranging from 68.1 to 96.9 ng/L, indicating also a possible center effect. 80.4% of the variation in the data was explained by the ANOVA model and the variable center, day, and interaction term accounted for 74.7%, 1.5% (non-significant [NS]), and 4.2% (NS), respectively. The same conclusions could be drawn from the observation of the results of pool 2.

For ELISA, even though there was a clear center effect for pool 1, it is accounted only for 14.1% of the variation. The majority of the variation was due to random error (47.2%) and day (22.9%). For pool 2, the variation due to the center effect was much larger (69.3%) compared to day (9.6%) and random error (9.1%).

#### Imprecision of the Methods

The imprecision of the methods according to the CLSI EP15-A3 guideline is presented in Table 1. Overall, coefficients of variation (CV) for the automated methods were better than the desirable intra-individual CV (7.6%) [8], except for GR on pool 1 on both Elecsys and iSYS assays. For the IDS ELISA, the CV was > 7.6% on both pools in DK and on pool 1 in BE. In GR, the CV was < 7.6% for both pools.

**Table 1:** Imprecision (CV%) Roche Elecsys, IDS-iSYS, and IDS ELISA of ß-CTX assays according to the CLSI EP15-A3 guideline

	Elecsys		IDS-iSYS		ELISA	
	Pool 1	Pool 2	Pool 1	Pool 2	Pool 1	Pool 2
ALL	**11.3**	**13.3**	**15.6**	**9.1**	**19.3**	**22.0**
BE	3.3	2.1	6.7	4.5	**8.8**	5.0
DK	2.9	3.5	6.1	2.0	**8.6**	**10.4**
GR	**8.2**	2.4	**12.2**	4.7	6.9	2.0
UK	7.0	4.6	7.2	2.5	NP	NP

The values in bold are those higher than the desirable coefficient of variation (7.6%), based on intra-individual variation of the biomarker

*NP* not performed

### Analysis of Patient Samples

#### Comparison Plasma vs. Serum

All Passing-Bablok regressions for comparisons between serum and plasma for each assay are presented in Table 2. The slope observed between plasma and serum for IDS-iSYS method was higher (1.15) compared with the other two methods (both at 1.02).

**Table 2:** Passing-Bablok correlation between ß-CTX run in plasma (y) and serum (x)

	Roche Elecsys	IDS-iSYS	IDS ELISA
ALL			
Slope (95% CI)	1.02 (1.01; 1.04)	1.15 (1.14; 1.17)	1.03 (1.01; 1.05)
Intercept (95% CI)	− 10.9 (− 14.1; − 7.5)	6.8 (4.2; 9.5)	− 10.1 (− 16.7; − 4.7)
n	778	794	567
BE			
Slope (95% CI)	1.04 (1.02; 1.07)	1.30 (1.27; 1.32)	1.04 (1.00; 1.08)
Intercept (95% CI)	− 12.5 (− 20.4; − 6.0)	4.8 (− 1.6; 10.7)	0.7 (− 18.0; 13.8)
n	200	200	190
DK			
Slope (95% CI)	0.95 (0.94; 0.97)	1.09 (1.06; 1.12)	0.98 (0.95; 1.00)
Intercept (95% CI)	− 7.1 (− 10.6; − 4.9)	16.1 (11.4; 22.1)	− 7.2 (− 10.0; − 1.7)
n	184	192	176
GR			
Slope (95% CI)	1.04 (1.02; 1.06)	1.17 (1.15; 1.19)	1.00 (0.95; 1.05)
Intercept (95% CI)	− 0.2 (− 8.0; 7.5)	− 12.7 (− 20.3; − 5.0)	10.4 (− 11.5; 32.8)
n	199	204	201
UK			
Slope (95% CI)	0.95 (0.93; 0.98)	1.13 (1.11; 1.15)	NP
Intercept (95% CI)	2.3 (− 2.7; 8.7)	2.9 (− 1.1; 6.0)	
n	195	200	

*NP* not performed

**Fig. 2:**
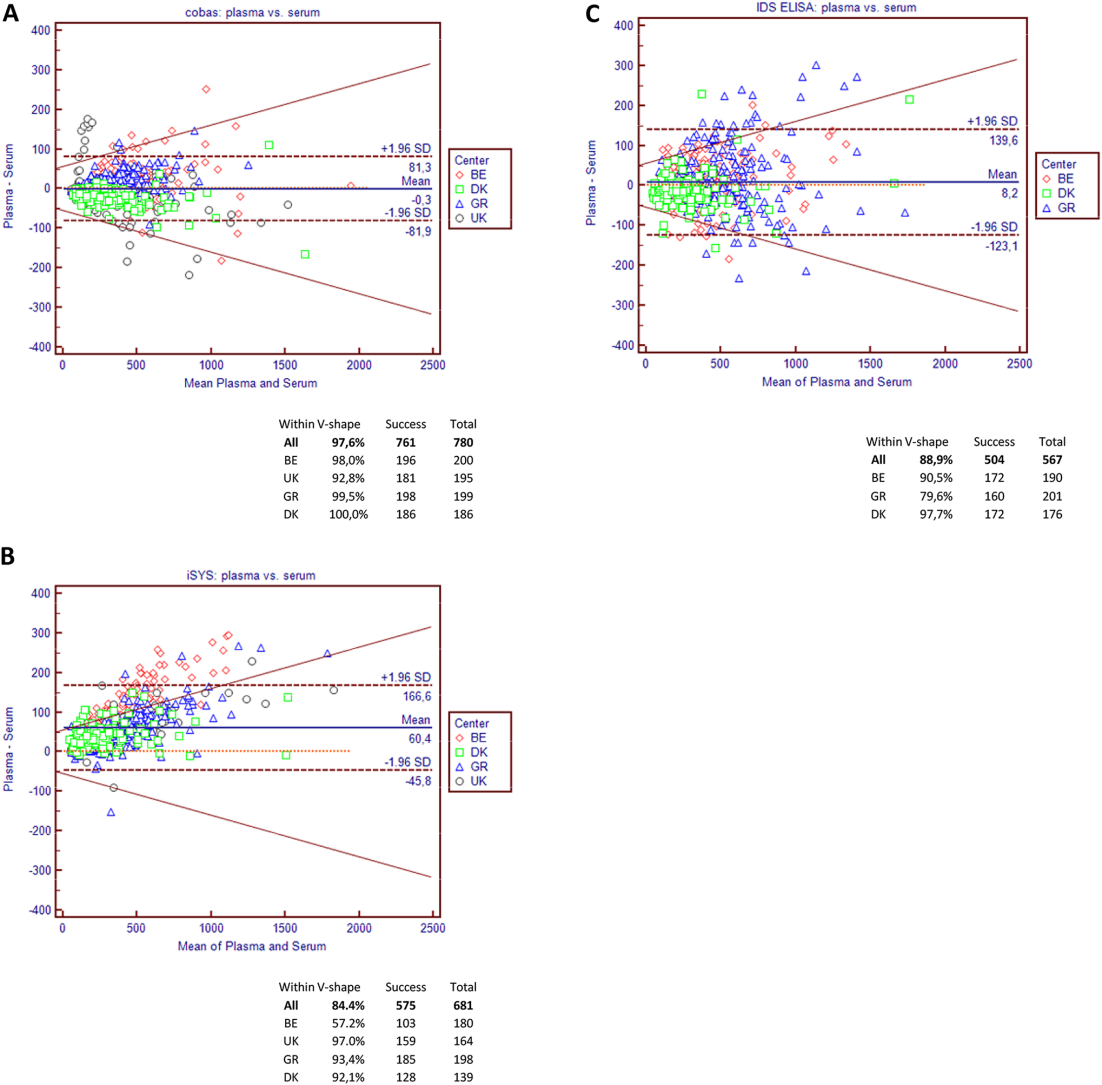
Bland–Altman plots and V-shape models comparing values observed in plasma vs. serum on Elecsys (**a**), iSYS (**b**), and ELISA (**c**)

For the Elecsys assay, the V-Shape model (Fig. 2a) shows that, overall, 97.6% of the observations fitted within the limits with very little center disparities (98.0% in BE, 92.8% in UK, 99.5% in GR and 100% in DK). With iSYS, the overall agreement was 84.4% (Fig. 2b), mainly due to the poor agreement observed in BE (57.2%), whereas the other three centers presented a much better concordance, > 90%. Of note, BE is the center where the samples have been stored for the longest period (up to 2 years) compared to the other three centers (< 6 months). For IDS ELISA (Fig. 2c), the results of the V-shape showed an overall agreement of 88.9% with some disparities between the centers (90.5% in BE, 79.6% in GR, and 97.7% in DK).

### Comparison of Methods in Plasma and Serum

#### Elecsys vs. IDS-iSYS

In plasma, the Passing-Bablok regression on the relationship between the Elecsys and iSYS assays was Roche Elecsys = 0.85*IDS-iSYS + 72.3 and in serum, it was Roche Elecsys = 0.97*IDS-iSYS + 96.1 (Table 3).

**Table 3:**
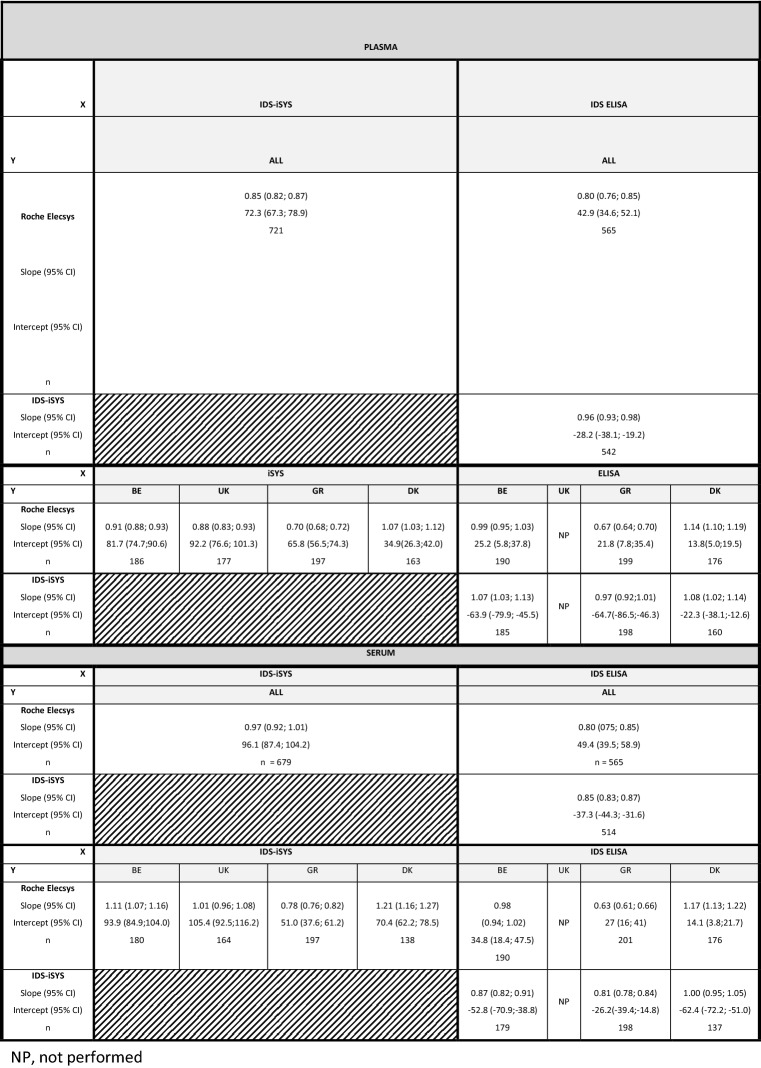
Passing-Bablok regression analyses of ß-CTX values performed with Roche Elecsys, IDS-iSYS, and IDS ELISA assays on serum and plasma specimens

The upper line of result corresponds to the slope (95% confidence interval) and the second line to the intercept (95% confidence interval)

*NP* not performed

**Fig. 3:**
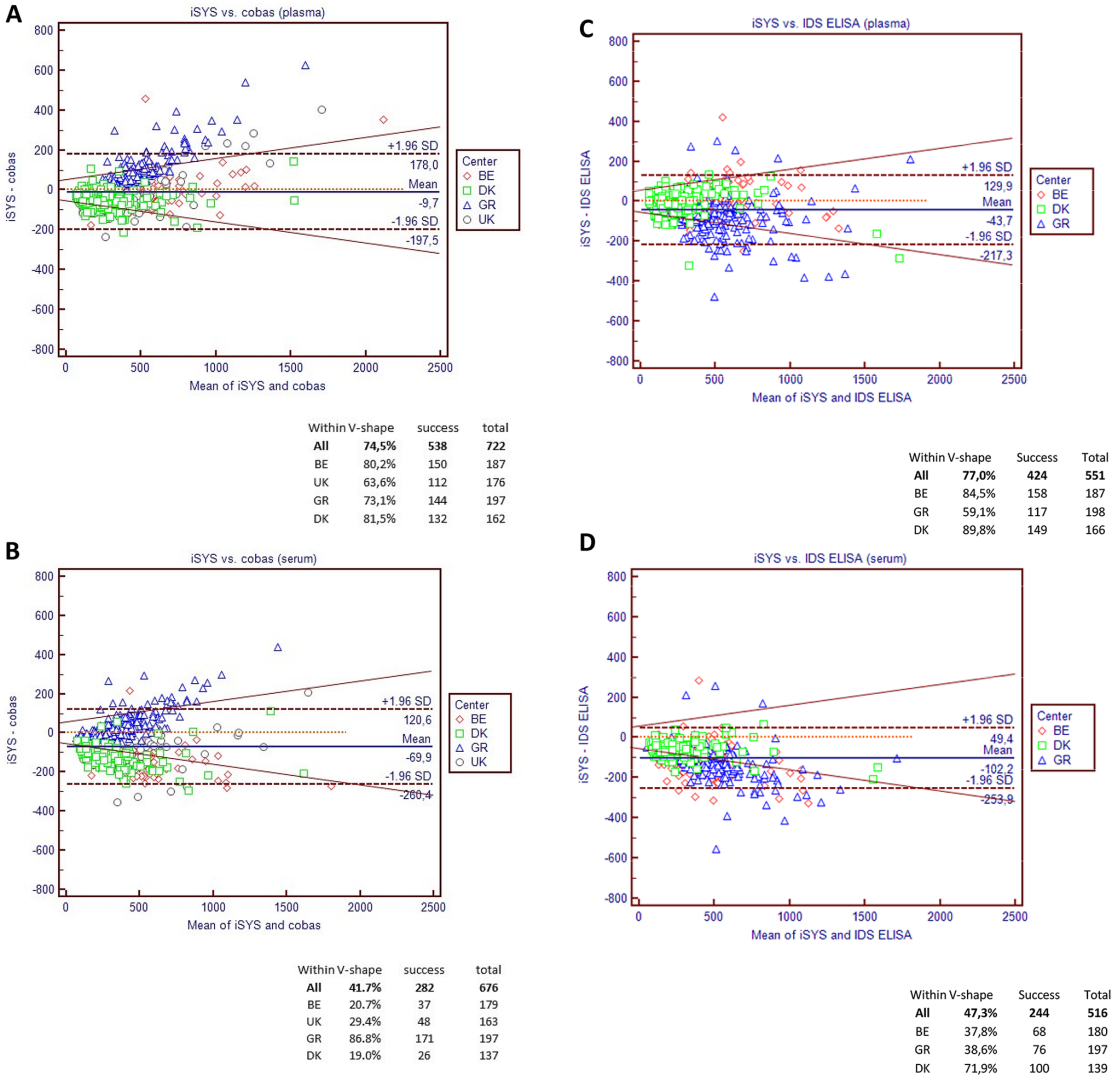

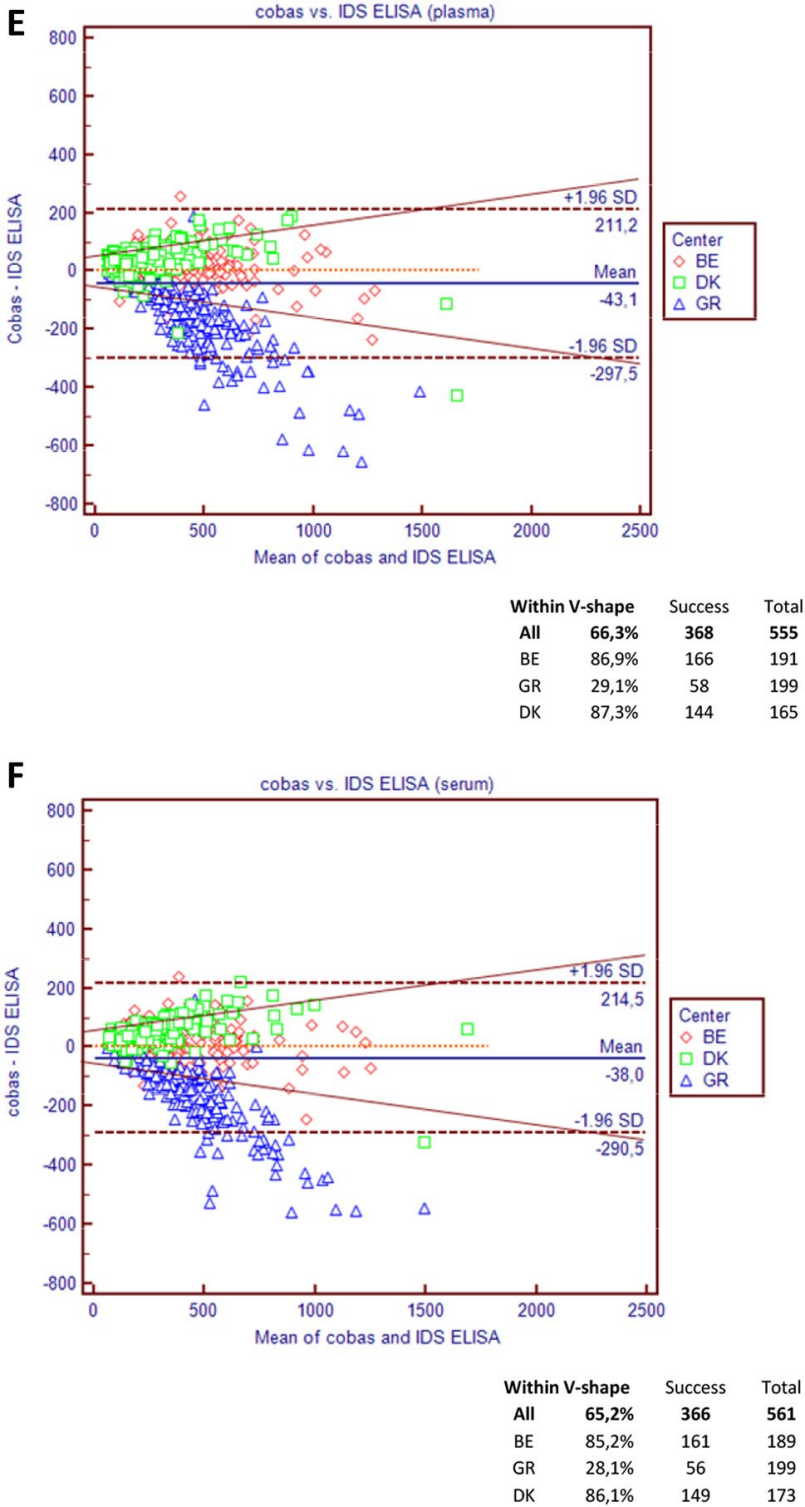
Bland–Altman and V-shape models comparing values observed in serum vs. plasma on iSYS vs. Elecsys (**a**, **b**), iSYS vs. IDS ELISA (**c**, **d**) and Elecsys vs. IDS ELISA (**e**, **f**). The bold dotted lines correspond to the bias ± 1.96 SD of the differences

The V-shaped model shows that, overall, 74.5% of the values were within the limits for plasma vs. 41.7% only for serum. Disparities between centers were important: for plasma, the percentage of samples comprised within the limits was 80.2%, 63.6%, 73.1%, and 81.5% for BE, UK, GR, and DK, respectively. It was even more marked in serum with 20.7%, 29.4%, 86.8%, and 19.0% of the samples from BE, UK, GR, and DK present in the defined limits, respectively (Fig. 3a, b).

In the center GR, the Bland Altman plots showed that the difference between the iSYS and Elecsys assays systematically increased with increasing ß-CTX concentrations in both plasma and serum (iSYS providing higher concentrations than Elecsys). This picture is very different from the three other centers, where iSYS generally gave lower values than the Elecsys assay. That was particularly true for serum.

#### IDS-iSYS vs. IDS ELISA

In plasma, the Passing-Bablok regression on the relationship between iSYS and ELISA was IDS-iSYS = 0.95*IDS ELISA-25, and in serum, it was iSYS = 0.84*IDS ELISA-37 (Table 3). The Bland–Altman plots (Fig. 3c, d) showed that iSYS generally gave lower ß-CTX values than the ELISA. That was specifically also true for serum where we observed a similar picture to what has been observed in serum when we compared the iSYS and Elecsys assays (see above) (Fig. 3c, d). The V-shape limits showed an overall agreement of 77.0% for plasma vs. 47.3% for serum with large disparities between centers: 84.5%, 59.1%, and 89.8% of the values were comprised in the limits in plasma for BE, GR, and DK, respectively, whereas they were of 37.8%, 38.6%, and 71.9% in serum for BE, GR, and DK, respectively.

#### Elecsys vs. IDS ELISA

In plasma, the Passing-Bablok regressions on the relationship between the Elecsys and ELISA assays was Roche Elecsys = 0.81*IDS ELISA + 43, and in serum, the relation was Elecsys = 0.80*IDS ELISA + 48 (Table 3). The Bland Altman plots (Fig. 3e, f) show a marked decrease in differences with increasing values in both media in GR, similarly to that observed when comparing Elecsys and iSYS. Centers BE and DK were very close in serum and plasma, presenting a slight increase in both media. This was confirmed by the V-shape limits showing percentages of samples between the limits of 86.9 and 85.2% in BE for plasma vs. serum compared to 87.3% and 85.2% in DK. The center GR had only 29.1% and 28.1% of the samples within the V-shape, which impacted the overall percentage of agreement (66.3 and 65.2% for plasma and serum, respectively).

To identify factors contributing to the variation between the ß-CTX values generated by the three assays, we calculated multivariable models with age, sex, center, BMI, weight, height, BMD, fasting status, and medication as independent covariates (data not shown). The two variables that appeared in all models were age and center, with center having the largest effect and age a significant but small effect.

Finally, the CCC showed that the degree of agreement between the methods was also center dependent and ranged from “poor” to “substantial.” Overall, the degree of agreement was better in plasma than in serum (Table 4). Indeed, in plasma, the degree of agreement was moderate for Elecsys vs. iSYS and for iSYS vs. ELISA and was poor for Elecsys vs. ELISA, whereas in serum, it was poor whatever the compared methods.

**Table 4:**
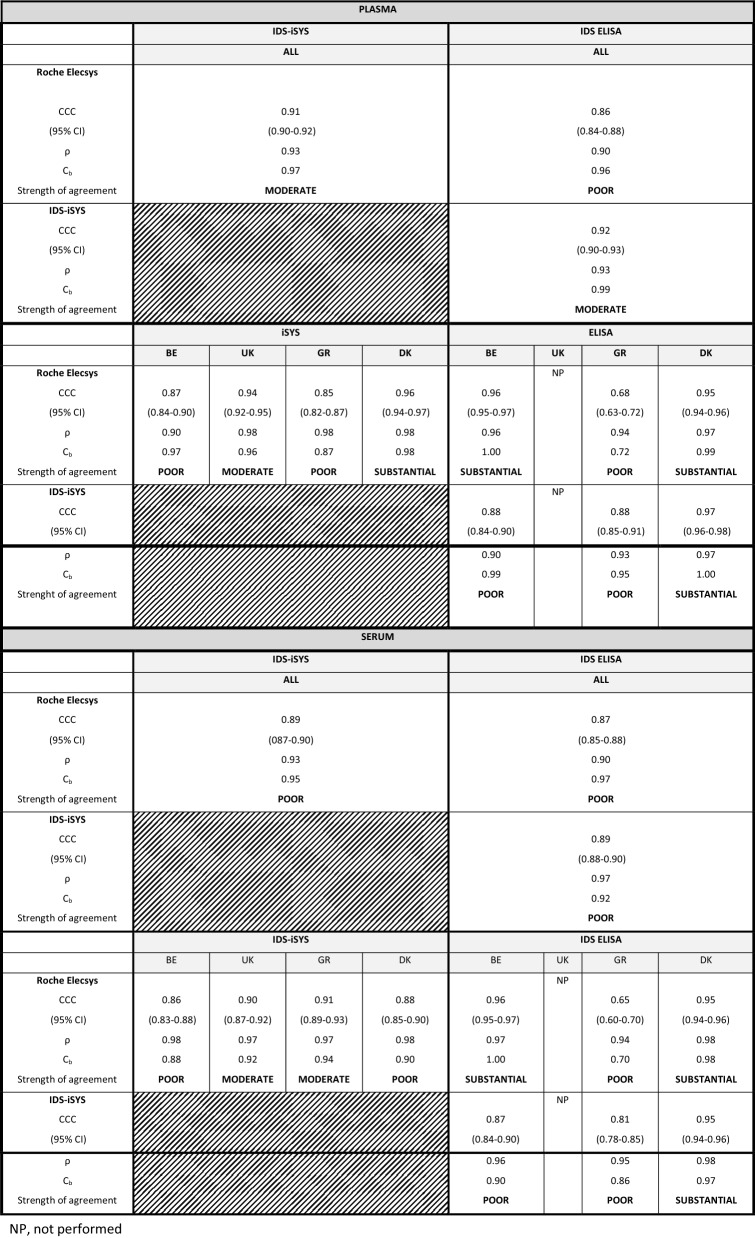
Concordance correlation coefficients (CCC), Pearson correlation coefficient (ρ), and bias correction factor (Cb)

*NP* no performed

### Analysis of Internal Quality Control Results

Each center was free to run the QC according to their routine procedure. There were 3 levels of QC for iSYS which were run 19 times in BE, 10 times in DK, 12 times in GR, and 5 times in UK. The 95% confidence intervals method (Tukey) shows that no significant difference was observed between BE, DK, and GR in any of the 3 controls, whereas UK presented lower values ranging from − 14 to − 8%. For the Elecsys assay, there were 2 levels of QC that were run 19 times in BE, 10 times in DK, 20 times in GR, and 5 times in UK. The 95% confidence intervals method shows that, for level 1, there was a non-significant trend for GR to have lower QC results, whereas for level 2, there was a significant difference of approximately 7% between BE and GR and between DK and GR.

## Discussion

Bone turnover markers, and notably bone resorption markers, are commonly used to monitor patients’ response to pharmacological treatment and adherence [2]. They have the great advantage, over bone mineral densitometry, in that they change rapidly following initiation of treatment, and these changes have suggested an association with reduction in fracture risk [11]. There would, thus, be a great benefit to follow patients with these markers, but many clinicians raise concerns before using them in clinical practice for the following reasons. First, these markers are very sensitive to pre-analytical variation including circadian rhythm and food intake [12]. Standardized procedures for specimen collection are, thus, mandatory for optimal patient follow-up. Second, reference ranges are not always correctly established and information on intra-individual variation is scarce. Third, there is variation between values generated by the different routine clinical assays due to non-availability of higher order reference methods (e.g., LCMS/MS methods) or internationally accepted reference standard material for the calibration of these assays. ß-CTX is a complex analyte in biological fluids, existing in type I collagen breakdown products ranging in molecular weight between 1000 and 10,000 Da [13]. For these reasons, IOF and IFCC have established a Committee for the standardization of bone marker assays, whose task was to standardize or harmonize (as technically feasible or appropriate at this time) clinical ß-CTX assays available for routine and research use, in serum and EDTA plasma.

Indeed, available data on method comparison for ß-CTX are scarce. An indirect comparison performed by one of us (RE) showed that upper reference limits were different when ß-CTX was measured in serum with the Roche Elecsys assay and the Nordic Biosciences ELISA (that later became IDS ELISA), ELISA providing a higher upper reference limit than Elecsys [14]. Similar data were shown in a recent comparison between the IDS-iSYS and the Roche Elecsys assays [15]. In a short communication, another one of us (EC) showed that the Passing-Bablok regression for serum ß-CTX with Roche Elecsys vs. IDS-iSYS assays was Roche Elecsys = 0.89*IDS-iSYS + 21.6 [16]. Chubb et al. evaluated ß-CTX values measured in EDTA plasma with the Roche Elecsys, IDS-iSYS, and IDS ELISA assays in 161 fasting men and women presenting to a bone clinic [6]. Comparing IDS-iSYS and Roche Elecsys, they found a huge proportional bias (0.62) and a systematic bias comparable to that reported here, while comparison with the IDS ELISA data yielded results that were more discrepant than the current data [6]. Wheater et al. also compared ß-CTX in sera of 72 self-reported healthy volunteers and 55 rheumatoid arthritis patients on Roche Elecsys and IDS-iSYS assays and found a large proportional bias of 1.29 (95% CI: 1.24; 1.34) and a constant bias of − 24 µg/L (95%CI: − 34; − 13) [17]. Finally, another of us (NRJ) recently compared 2308 serum ß-CTX levels measured with the IDS-iSYS and Roche Elecsys assays on samples stored at − 80 °C for < 2 years. The regression equation was CTX IDS-iSYS = 1.39*CTX Roche Elecsys − 139.75 [18].

In this study, we have reported the Passing-Bablok regressions equations for ß-CTX values in serum and plasma specimens for each of the routine clinical ß-CTX assays currently available and for comparisons of each of the assays in both serum and plasma. We have also compared the methods using Bland–Altman plots and V-shapes limits for the regression of differences on averages.

Our results show substantial variation between methods. Specifically, the largest variations were observed for

1. Serum vs. plasma for iSYS in BE and
2. Elecsys vs. iSYS and ELISA in GR.

To further explore these variations, 80 patients samples (serum and EDTA plasma), stored in GR at − 80 °C were reanalyzed in GR in 2019 on the cobas® platform by the Elecsys assay and shipped to BE where they were reanalyzed on cobas, together with 80 samples stored in BE. Compared to the results obtained in 2017, the Passing-Bablok regression of the Elecsys plasma values of the 80 BE patients was CTX (2017) = 1.11 × CTX (2019)—7 whereas the Passing-Bablok of the 80 GR patients rerun in GR was CTX (2017) = 0.90 × CTX (2019)—9. The Passing-Bablok of the GR samples run in GR and rerun in BE (both in 2019) was GR = 1.00 × BE—1.2. Long-term stability studies on ß-CTX are scarce. One study has shown that no decrease could be observed in concentrations after 3 years storage at − 80 °C [19]. However, if we assume that long-term storage of samples and two cycles of freeze thawing can only lead to some slight decrease in the concentration of the analyte, this new set of results shows that the results obtained on the cobas instrument in GR in 2017 were probably falsely low, even if the curves of the cobas and the quality controls of the company were within the specified limits.

Compared to 2017, the 2019 ß-CTX values observed on the BE samples decreased by 13.5% and 8.7% on the cobas analyzer in serum and plasma, respectively. On iSYS, the percentage of decrease of ß-CTX in plasma samples was rather similar (− 7.5% and − 8.4% for the BE and GR samples, respectively). However, the decrease of values obtained on serum with iSYS was much larger: 27.6% and 22.6% for BE and GR samples, respectively. The reason why ß-CTX decreased more in serum on iSYS are unclear but may be linked to the configuration of the assay. Indeed, the iSYS assay uses a pair of monoclonal antibodies targeted against the octapeptide, whereas Elecsys and IDS ELISA kits use a pair of polyclonal/monoclonal antibodies. The difference in pH of stored serum and plasma samples (mean pH of a subset of 10 of these samples was 7.29 for serum and 8.06 for EDTA plasma) and/or inactivation of proteases by chelation of divalent cations by EDTA and/or pH could lead to a change in the conformation of the peptide. This change could be detected by the monoclonal/monoclonal sandwich but not by the polyclonal/monoclonal one. Since BE samples have been stored for a much longer period compared to GR, UK, and DK counterparts, the larger difference between plasma and serum observed on iSYS in the BE center could, thus, be due to this stability issue. Herrmann et al. have shown that at a pH > 8.0, serum ß-CTX concentration (measured by the Elecsys assay) remained relatively stable for several(?) days if samples were stored at + 4 °C [20]. Later on, Qvist et al. evaluated long-term ß-CTX stability at lower temperatures [19]. They used the Elecsys and the former Nordic Biosciences ELISA assay that uses a pair of monoclonal antibodies similarly to the IDS-iSYS. As already mentioned, these authors did not find any degradation of ß-CTX after 3 years of storage at − 80 °C, contrary to us, with any of the 2 kits. It must be noted, however, which the pH they observed in serum was much more basic than what we found. These discrepancies deserve some more investigations.

Imprecision also varied among the sites, particularly with regard to the ELISA method suggesting a higher degree of operator dependency for the manual assay.

The CCC is also an interesting way to compare methods. In this study, the CCC is better in plasma vs. serum and varied according to the center for the same method. Even if far from perfect, these results are, however, in line with what can be expected from another biomarkers, like 25(OH)D for instance [21, 22].

The strengths of this study are the multicenter approach, the large number of participants, the use of serum and plasma, and the robustness of the pre-analytical and analytical methods. Even though we attempted to harmonize the criteria for recruitment of patients, not all were fasting at the time of specimen collection, which might be perceived as a weakness of our study. It is known that fasting reduces the within-individual biological variability of ß-CTX [23]. However, this study was purely an analytical comparison of methods, and no follow-up of the patients was performed. We have identified that the relationship between the Elecsys and iSYS ß-CTX values is not significantly different between GR and BE (all fasting) and DK and UK (not all fasting), which suggests that food intake does not appear to have any impact on the relationship between the ß-CTX values produced by the various assays.

In conclusion, we report the results of a multicenter evaluation of ß-CTX with the current assays used in clinical laboratories and have derived regression equations for the interconversion of ß-CTX results assayed on serum and plasma specimens and between Roche **cobas e** and IDS-iSYS immunoassay platforms or ELISA plates. We identified 1. significant variation between the individual centers, each of whom is experienced with running these assays in clinical practice. Unfortunately, no useful regression equation could be calculated to harmonize results obtained with the different platforms, mainly because of the large between-center variations. 2. Our results reinforce our previous recommendation on the use of EDTA plasma over serum (especially in large epidemiological studies and therapeutic trials where the recruitment may be very long), and we recommend that patients are followed by the same method. For that purpose, we also recommend that laboratories identify the assay used for ß-CTX determination on their protocols.
