# Correction to: A Multicenter Study to Evaluate Harmonization of Assays for C-Terminal Telopeptides of Type I Collagen (ß-CTX): A Report from the IFCC-IOF Committee for Bone Metabolism (C-BM)

**DOI:** 10.1007/s00223-021-00839-y

**Published:** 2021-04-17

**Authors:** E. Cavalier, R. Eastell, N. R. Jørgensen, K. Makris, S. Tournis, S. Vasikaran, J. A. Kanis, C. Cooper, H. Pottel, H. A. Morris, on behalf of the IFCC-IOF Committee for Bone Metabolism (C-BM)

**Affiliations:** 1 Department of Clinical Chemistry, University of Liège, CHU Sart-Tilman, Domaine du Sart-Tilman, B-4000 Liège, Belgium; 2 Mellanby Centre for Bone Research, University of Sheffield, Sheffield, UK; 3 Department of Clinical Biochemistry, Rigshospitalet, 2600 Glostrup, Denmark; 4 Department of Clinical Medicine, Faculty of Health and Medical Sciences, University of Copenhagen, Copenhagen, Denmark; 5 Clinical Biochemistry Department, KAT General Hospital, 14561 Athens, Greece; 6 Laboratory for Research of the Musculoskeletal System “Th. Garofalidis”, Medical School, University of Athens, 14561 Athens, Greece; 7 PathWest Laboratory Medicine, Fiona Stanley Hospital, Murdoch, WA 6150 Australia; 8 Centre for Metabolic Bone Diseases, University of Sheffield Medical School, Beech Hill Road, Sheffield, S10 2RX UK; 9 The MRC Epidemiology Resource Centre, Southampton General Hospital, University of Southampton, Southampton, SO16 6YD UK; 10 Department of Public Health and Primary Care, KU Leuven Campus Kulak Kortrijk, Kortrijk, Belgium; 11 School of Pharmacy and Medical Sciences, University of South Australia, Adelaide, SA 5000 Australia; 12 Mary McKillop Institute for Health Research, Australian Catholic University, Melbourne, Australia

## Correction to: Calcified Tissue International 10.1007/s00223-021-00816-5

The original version of this article unfortunately contained a mistake in Fig. 1. The corrected Fig. 1 is given below.

**Fig. 1:**
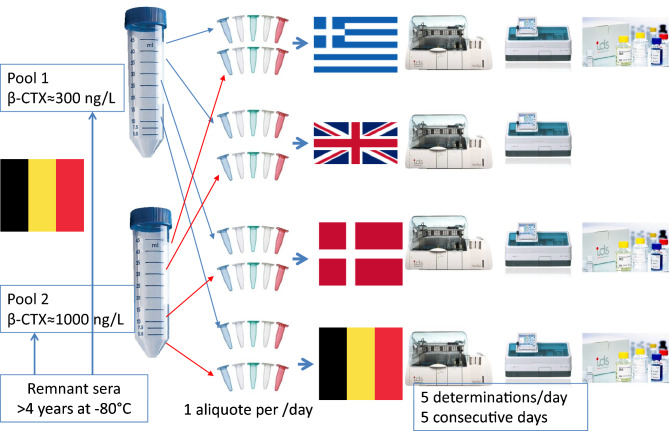
Preparation of the pools for the performance study evaluation

